# Homozygous *SPAG6* variants can induce nonsyndromic asthenoteratozoospermia with severe MMAF

**DOI:** 10.1186/s12958-022-00916-3

**Published:** 2022-03-01

**Authors:** Chuan Xu, Dongdong Tang, Zhongmei Shao, Hao Geng, Yang Gao, Kuokuo Li, Qing Tan, Guanxiong Wang, Chao Wang, Huan Wu, Guanjian Li, Mingrong Lv, Xiaojin He, Yunxia Cao

**Affiliations:** 1 Reproductive Medicine Center, Department of Obstetrics and Gynecology, the First Affiliated Hospital of Anhui Medical University, No 218 Jixi Road, Hefei, 230022 Anhui China; 2 NHC Key Laboratory of Study On Abnormal Gametes and Reproductive Tract (Anhui Medical University), No 81 Meishan Road, Hefei, 230032 Anhui China; 3 Key Laboratory of Population Health Across Life Cycle (Anhui Medical University), Ministry of Education of the People’s Republic of China, No 81 Meishan Road, Hefei, 230032 Anhui China; 4 Anhui Province Key Laboratory of Reproductive Health and Genetics, No 81 Meishan Road, Hefei, 230032 Anhui China; 5 Biopreservation and Artificial Organs, Anhui Provincial Engineering Research Center, Anhui Medical University, No 81 Meishan Road, Hefei, 230032 Anhui China

**Keywords:** MMAF, Gene, *SPAG6*, Asthenoteratozoospermia, ICSI

## Abstract

**Background:**

Multiple morphological abnormalities of the sperm flagella (MMAF) is a subtype of severe asthenoteratozoospermia with poorly understood genetic etiology. SPAG6 is a core axonemal component that plays a critical role in the formation of cilia and sperm flagella. Previous studies have reported that mutations in *SPAG6* cause primary ciliary dyskinesia (PCD), but the association between *SPAG6* gene variants and the MMAF phenotype has not yet been described.

**Methods:**

We performed whole-exome sequencing (WES) in two unrelated Han Chinese men with MMAF. Sanger sequencing was used to validate the candidate variants. Routine semen analysis was carried out according to the WHO guidelines (5^th^ Edition). Sperm morphology was assessed using modified Papanicolaou staining. Scanning and transmission electron microscopy (S/TEM) was performed to observe the ultrastructural defects of the sperm flagella. Western blot analysis and immunofluorescence (IF) of spermatozoa were performed to examine the expression of SPAG6 protein. Assisted fertilization with intracytoplasmic sperm injection (ICSI) was applied.

**Results:**

Two homozygous *SPAG6* variants were identified by WES and Sanger validation in two patients with MMAF phenotype (F1 II-1: c.308C > A, p. A103D; F2 II-1: c. 585delA, p. K196Sfs*6). Semen analysis showed progressive rates of less than 1%, and most of the spermatozoa presented MMAF by Papanicolaou staining. TEM revealed that the overall axonemal ultrastructure was disrupted and primarily presented an abnormal “9 + 0” configuration. No other PCD-related symptoms were found on physical examination and medical consultations, as well as lung CT screening. The level of SPAG6 protein was significantly decreased in the spermatozoa, and IF analysis revealed that SPAG6 staining was extremely weak and discontinuous in the sperm flagella of the two patients. Notably, F1 II-1 and his wife conceived successfully after undergoing ICSI.

**Conclusions:**

Our research provides new evidence for a potential correlation between *SPAG6* variants and the MMAF phenotype.

**Supplementary Information:**

The online version contains supplementary material available at 10.1186/s12958-022-00916-3.

## Background

Infertility affects 8–15% of couples who are trying to conceive, and has become a growing worldwide problem. Nearly half of all cases of infertility are attributed to male factors [1, 2]. Asthenoteratozoospermia is one of the most common factors leading to male infertility, and is characterized by poor sperm motility and obvious sperm morphological abnormalities [3, 4]. As a subtype of asthenoteratozoospermia, multiple morphological abnormalities of the sperm flagella (MMAF) manifests as varied flagellar malformations, including short, coiled, bent, absent, and/or irregular flagella, and results in severely impaired sperm motility [5]. The absence of central microtubules is considered a hallmark of the MMAF phenotype [6].

Bi-allelic mutations in *DNAH1* (MIM: 603,332) associated with MMAF were first described in 2014 [5]. To date, several genes responsible for MMAF have been identified, including *DNAH8* (MIM: 603,337), *SPEF2* (MIM:610,172), *ARMC2*(MIM:618,424), and *WDR19* (MIM:608,151) [7–10]. Mutations in these genes can disorganize microtubule assembly, contributing to the abnormal formation of the sperm flagella. However, the reported monogenic causes can only account for approximately 35% to 60% of MMAF cases [11, 12], suggesting that this phenotype has strong genetic heterogeneity and that further genetic exploration is needed.

The sperm-associated antigen 6 (*SPAG6*; MIM: 605,730) gene encodes an axonemal protein that plays a critical role in axonemal structural integrity and function [13]. *SPAG6* is primarily expressed in the lung and testis, and its variants are associated with multi-system dysfunctions involving cilia and flagella [14, 15], including primary ciliary dyskinesia (PCD). PCD is a rare multisystemic dysfunction caused by cilial motility malfunction. A recent study reported that bi-allelic mutations in *SPAG6* are related to PCD, accompanied by recurrent respiratory tract infections and male infertility [14]. Similarly, the phenotype of *Spag6*^−/−^ model mice is similar to that of some PCD-associated patients, including hydrocephalus and infertility resulting from a lack of motility related to ependymal cilia and sperm flagella [15]. These studies have demonstrated that mutations in *SPAG6* are a genetic factor leading to syndromic asthenozoospermia, including PCD. However, the potential relationship between *SPAG6* and nonsyndromic asthenoteratozoospermia characterized by the MMAF phenotype has not yet been described.

In this study, we performed whole-exome sequencing (WES) in two unrelated Han Chinese men affected with severe asthenoteratozoospermia, and homozygous variants in *SPAG6* were identified in both cases. Notably, the two *SPAG6*-mutated probands consistently exhibited a typical MMAF phenotype and no other PCD-related symptoms. These results provide new evidence for a potential correlation between *SPAG6* variants and nonsyndromic asthenoteratozoospermia characterized by the MMAF phenotype.

## Methods

### Subjects and clinical investigation

Two unrelated Han Chinese men from consanguineous families and diagnosed with primary infertility were recruited from the Reproductive Center of the First Affiliated Hospital of Anhui Medical University (Hefei, China), and were enrolled in the context of severe asthenoteratozoospermia with a representative MMAF phenotype. All subjects received detailed medical consultations and excluded other related risk factors, including abnormal chromosomal karyotypes, Y chromosome microdeletions, abnormal levels of sex hormones, radiotherapy, and chemotherapy. Physical examination revealed normal external genitalia and bilateral testicles, with no obvious abnormalities in the bilateral spermatic veins. Two healthy men with normal fertility and normal semen characteristics served as the control group. Peripheral whole blood from each individual was collected for subsequent genetic analysis. This research was reviewed and approved by the ethics board committee of the First Affiliated Hospital of Anhui Medical University, and all individuals provided written informed consent.

### WES and Sanger sequencing

For WES and bioinformatic analysis, genomic DNA was extracted from whole peripheral blood samples and the exome was enriched using the SureSelect XT Human All Exon Kit (Agilent). Details of the study protocol have been published in our previous study [16]. Briefly, we extracted DNA from whole peripheral blood of the patients and sequenced with the MGISEQ-2000 platform. The data were mapped to the hg19 and GATK were used to call genetic variants. We annotated variants using allele frequency databases (the 1000 Genomes Project, gnomAD, and ExAC) and deleterious prediction tools. The variant with allele frequencies > 0.01 were excluded. We focused on loss-of-function variants including stop gain/loss, frameshift insertion/deletion, splicing within two base pairs, and potentially deleterious missense variants that were predicted to be deleterious by two of the three tools (PolyPhen-2, SIFT, and Mutation Taster). The pathogenic variants in testis highly expressed genes were used for further analysis. We screened putative bi-allelic variants or X-linked variants of the patients according to the recessive or X-linked model of MMAF. Due to the consanguineous status and the low prevalence of MMAF, we mainly focused on autosomal homozygous variants or X-linked variants. Sanger sequencing was used to validate the inheritance patterns. The pathogenic variants were also examined using WES for the respective female partners. Primers used for Sanger sequencing are listed in Supplementary Table 1.

### Semen analysis and sperm morphology

Routine semen analysis was carried out using the Sperm Class Analyzer (SCA) 5.1 version software (Microptic, Spain), according to the World Health Organization guidelines (5th Edition) [17]. Sperm morphology was assessed using modified Papanicolaou staining. At least 200 spermatozoa from each participant were examined to assess defects in sperm flagella. The MMAF phenotype was classified into five categories: (1) absent, (2) short, (3) angulation, (4) coiled, and (5) irregular caliber flagella [5].

### Scanning and transmission electron microscopy

For scanning electron microscopy (SEM) and transmission electron microscopy (TEM), sperm samples were collected and fixed with 2.5% glutaraldehyde at 4 °C for at least 2 h. For SEM, after the dehydration step, the samples were dried chemically using hexamethyldisilazane, and then added dropwise to the specimen stubs. For TEM, samples were embedded in Epon, and ultrathin sections were cut and stained with uranyl acetate and lead citrate. Images were obtained with a Nova Nano 450 (Thermo Fisher, USA) and a Tecnai G2 Spirit BioTWIN (FEI, USA) electron microscope, respectively.

### Western blotting and immunofluorescence staining

Western blot analysis of spermatozoa was performed according to a previously described protocol [18]. The primary antibody was rabbit polyclonal anti-SPAG6 (HPA038440, Sigma, USA) antibody, and protein expression levels were normalized to that of beta-actin.

Immunofluorescence (IF) analysis was conducted to examine changes in the SPAG6 protein in the spermatozoa, according to our previously published protocol [19]. The primary antibodies were rabbit polyclonal anti-SPAG6 (HPA038440, Sigma, USA) and mouse monoclonal anti-acetylated tubulin (5335S, Cell Signaling Technology, USA). Fluorescence images were captured using an LSM800 laser scanning confocal microscope (Zeiss, Germany).

### Assisted reproductive procedures

Standard controlled ovarian hyperstimulation was performed according to our previous publication [20]. To enrich the motile spermatozoa, semen samples were processed by discontinuous density gradient centrifugation. After oocyte retrieval, mature oocytes and motile spermatozoa were selected for intracytoplasmic sperm injection (ICSI). All embryos formed after standard embryo culture were cryopreserved via vitrification for the following frozen-thawed cycles. After two months, either one or two viable blastocysts were thawed and transferred to the uterus of the female partner. Serum β-HCG levels were measured on day 14 after embryo transfer to determine biochemical pregnancy, and clinical pregnancy was defined as the presence of fetal heart activity in utero confirmed by B-ultrasound 30 days after embryo transfer.

## Results

### Two homozygous SPAG6 gene variants were identified in men with MMAF

**Fig. 1:**
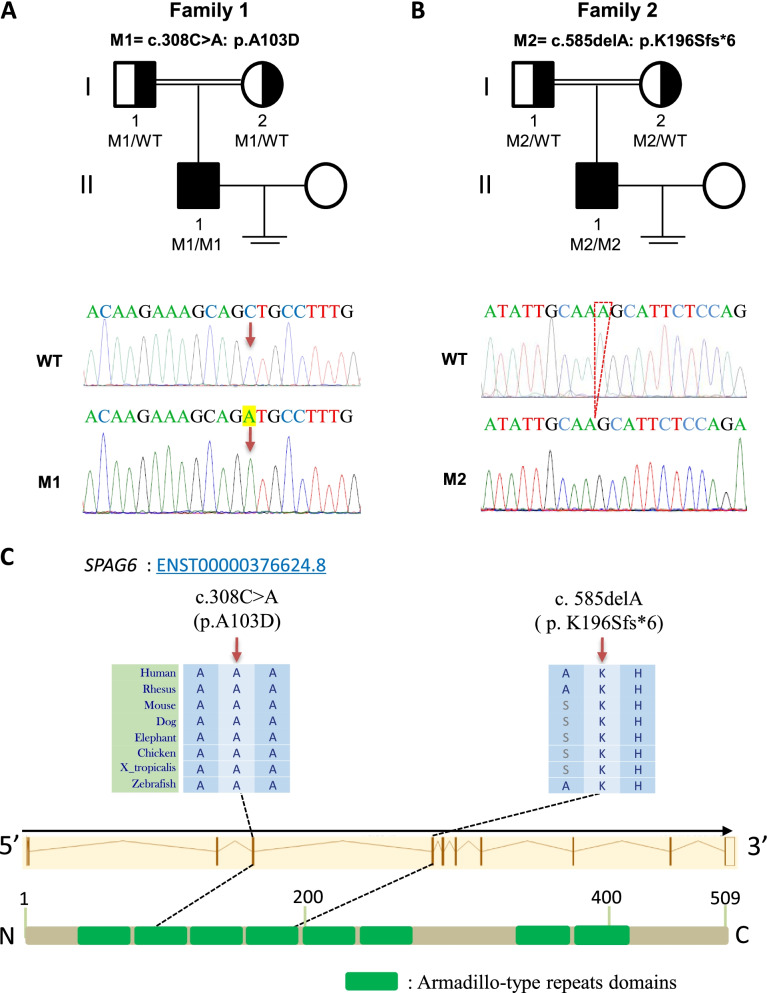
Variants of *SPAG6* in the two patients with MMAF from two consanguineous families. **A**-**B** Pedigrees of the two families affected by the variants in *SPAG6*. The brown arrow and red dotted line show mutated locations in the validation of Sanger sequencing. **C** a schematic diagram of mutated positions occurred in the SPAG6 protein. The mutated positions of *SPAG6* are conserved among species. The green boxes indicate the armadillo-type repeats domains of SPAG6 protein. WT, wild type; M, *SPAG6* mutations; MMAF: multiple morphological abnormalities of the sperm flagella

To probe the genetic factors contributing to the MMAF phenotype, we analyzed the two probands by WES. Bioinformatics analysis was applied as mentioned in method section, and relevant or meaning mutations were remained (Supplementary Table 2). After bioinformatic filtering, the two probands were found to harbor homozygous variants of *SPAG6*, the only gene related to sperm flagellar function that met the screening conditions. Following Sanger sequencing, homozygous *SPAG6* variants were identified in two individuals and inherited from their heterozygous parental carriers. The details are summarized in the Fig. 1 and Table 1.

**Table 1:** Genetic information of *SPAG6* variants of the patients

Subject	F1 II-1	F2 II-1
**cDNA mutation**	c.308C > A	c. 585delA
Exon	Exon 4	Exon 5
Mutation type	nonsynonymous	Frameshift deletion
Protein alteration	p. Ala103Asp	p. Lys196Serfs*6
**Allele frequency in human population**		
1KGP	NA	NA
ExAc_all	NA	8.25 x 10^-6^
gnomAD	NA	4.07 x 10^-6^
**Deleterious prediction**		
SIFT	D	NA
PolyPhen-2	D	NA
Mutation Taster	D	NA

RefSeq accession number of *SPAG6* is NM_ 012,443.4

*Abbreviations*: *1KGP* 1000 Genomes Project, *ExAc_all* all the data of Exome Aggregation Consortium *gnomAD* the Genome Aggregation Database, *D* disease-causing, *NA* not available

### Sperm analysis and clinical characteristics of men carrying SPAG6 variants

Sperm parameters are summarized in Table 2. Semen analysis of the patients showed severe asthenoteratozoospermia with progressive motility rates of less than 1%, while the semen volume, sperm concentration, and sperm vitality were normal. Clinical examinations were also performed. None of the patients suffered from PCD-related symptoms, such as chronic bronchitis, recurrent respiratory tract interference, otitis media, and visceral inversion (Supplementary Table 3). Lung CT imaging further confirmed normal results in both cases (Supplementary Fig. 1). The MMAF phenotype in the spermatozoa was examined using light microscopy. According to the Papanicolaou staining method, most of the spermatozoa presented multiple flagellar malformations, including absent, short, coiled, angulation, and irregular flagella. Notably, short and coiled flagella were most frequently observed in the spermatozoa of the two patients.

**Table 2:** Semen parameters and sperm morphology of men harbouring homozygous *SPAG6* variants

**Subject**	**F1 II-1**		**F2 II-1**		**Reference Values**^a^
**Age**	29		26		
**Semen parameter**	Sample 1	Sample 2	Sample 1	Sample 2	
Semen volume (mL)	2.2	3.5	3.4	3.6	> 1.5
Concentration (10^6^/mL)	107.0	63.9	16.8	27.4	> 15.0
Motility (%)	**2.0**	**2.7**	**0**	**0**	> 40.0
Progressive motility (%)	**0.3**	**0.5**	**0**	**0**	> 32.0
Viability (%)	66	NA	61	NA	> 58.0
**Sperm Morphology**					
Normal flagella (%)	3.2		4.1		> 23.0
Absent flagella (%)	4.5		7.1		< 5.0
Short flagella (%)	**55.3**		**47.6**		< 1.0
Coiled flagella (%)	**32.5**		**39.2**		< 17.0
Angulation (%)	3.5		1.5		< 13.0
Irregular caliber (%)	1.0		0.5		< 2.0

*Abbreviations*: *NA* Not applicable

^a^Reference Values according to the WHO (2010) criteria; Bold characters represent abnormal values

**Fig. 2:**
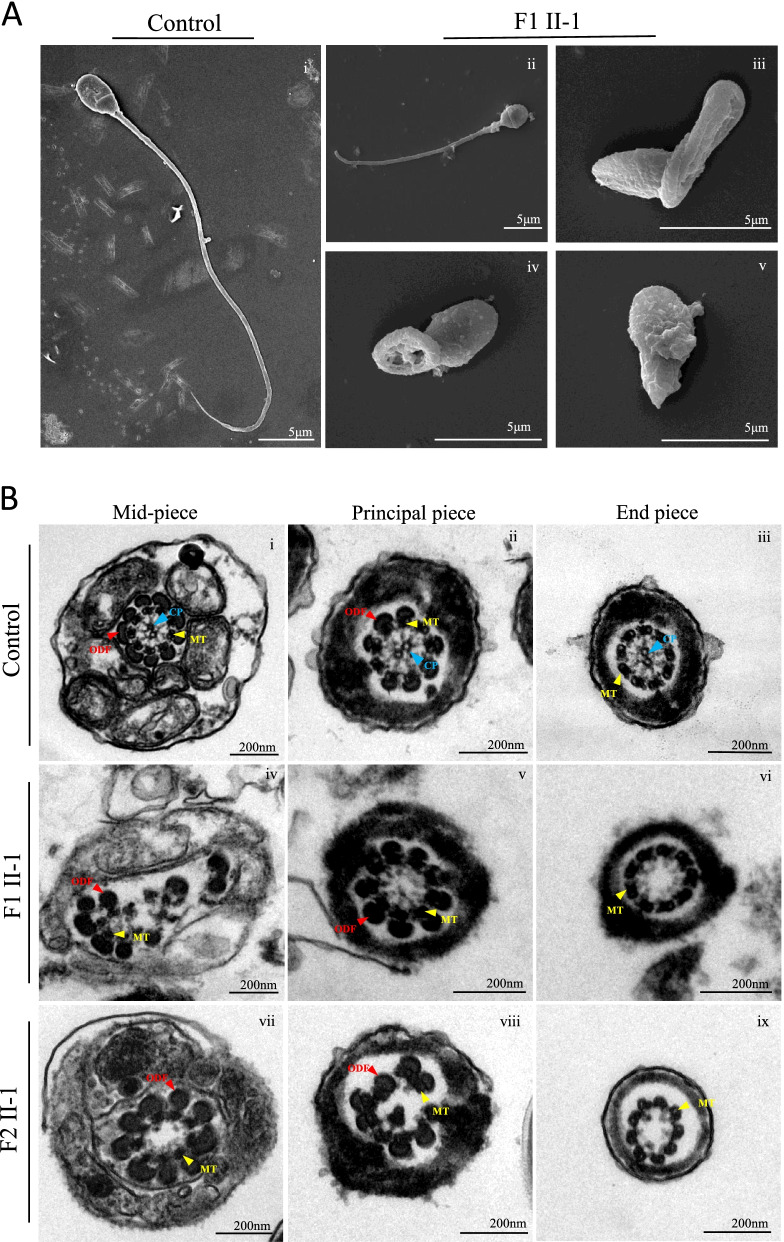
Ultrastructural defects in spermatozoa from two individuals carrying *SPAG6* variants. **A** Scanning electron microscopy of the spermatozoa from a healthy control and F1 II-1. (i) normal morphology of spermatozoa from a healthy control man; (ii-v) scanning electron microscopy showed the multiple abnormalities of the sperm flagella from F1 II-1 individual, including short(ii), coiled (iii, iv), and absent(v). Scale bar: 5 μm. **B** transmission electron microscopy of the sperm flagella from a healthy control and two patients. (i-iii) cross-sections of the sperm flagella in a healthy control, including mid-piece, principal piece and end-piece, show the typical “9 + 2” axonemal structure and peri-axoneme structure. (iv-vi) and (vii-ix) axonemal cross-sections of the sperm flagella from F1 II-1 and F2 II-1, respectively. The overall axonemal ultrastructure was disrupted, and primarily presented an abnormal “9 + 0” configuration. Of these, the lack of the central microtubules was the main defect observed in sperm flagella from two probands, peripheral microtubule doublets and outer dense fibers were also translocated and disorganized. Scale bar: 200 nm. Abbreviations: CP, central pair of microtubules (blue triangles); MT, peripheral microtubule doublets (yellow triangles); ODF, outer dense fiber (red triangles);

To further reveal ultrastructural defects in the flagella, we also used S/TEM to analyze the spermatozoa from patients and controls. According to SEM, the spermatozoa of the patients primarily presented short and coiled flagella (Fig. 2A). For TEM, over 50 random flagellar cross-sections were observed for each *SPAG6*-mutated proband to observe the microtubule assembly in the sperm flagella. In contrast to the typical “9 + 2” configuration that was observed in the normal controls, the overall axonemal ultrastructure was disrupted, and primarily presented an abnormal “9 + 0” configuration in sperm flagella of both patients. Of these, the lack of the central microtubules was the main defect observed in sperm flagella, additionally, peripheral microtubule doublets and outer dense fibers were also translocated and disorganized (Fig. 2B).

### Expression of SPAG6 was attenuated in spermatozoa from men with MMAF

**Fig. 3:**
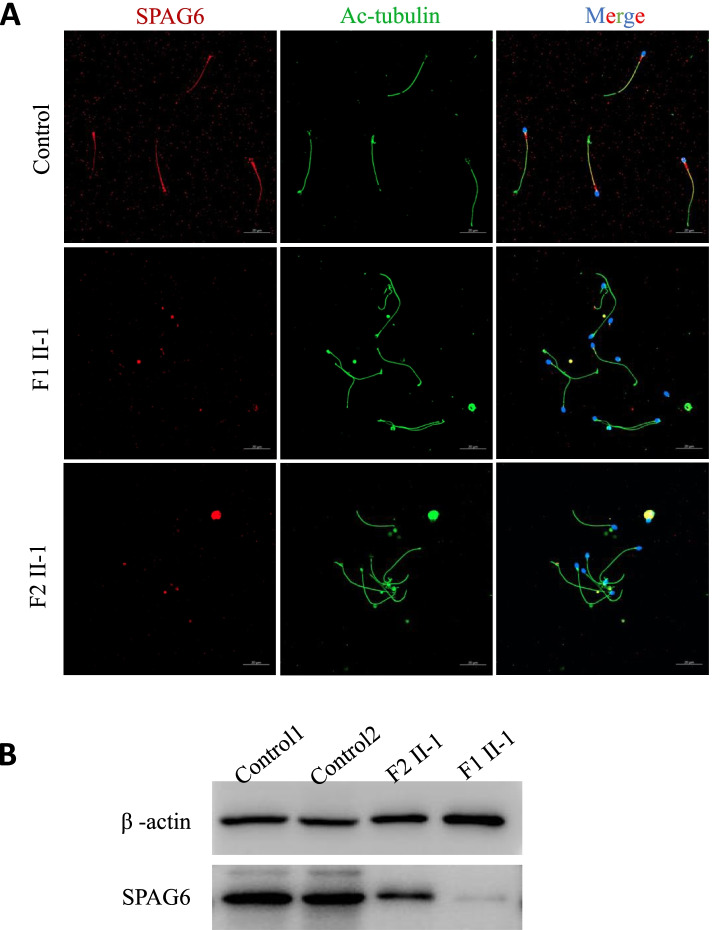
Lower expression of SPAG6 in spermatozoa from men harboring *SPAG6* variants. **A** Immunofluorescence analysis: SPAG6 staining (red) was located along entire the sperm flagella from a normal control, while SPAG6 staining was extremely weak and discontinuous in the sperm flagella from F1 II-1 and F2 II-1. The anti-acetylated tubulin staining (green) was used as a flagellar maker. Scale bar: 20 µm. **B** SPAG6 protein levels were determined using western blotting in spermatozoa from F1 II-1, F2 II-1 and two healthy controls. Beta-actin was used as loading control

To further investigate the pathogenicity of the identified homozygous *SPAG6* variants, expression of SPAG6 protein was analyzed in spermatozoa from the controls and two cases. The western blot showed an intense band at approximately 55 kDa for the full-length SPAG6 protein in the normal control, and the intensity of this band was dramatically reduced in the two probands (Fig. 3B). The localization and expression of SPAG6 protein in spermatozoa was also detected by IF staining. SPAG6 staining was localized along the entire flagella of sperm from the controls, and normal axonemes were visible via staining of acetylated-α-tubulin. In contrast, SPAG6 staining was extremely weak and discontinuous in sperm flagella in both of the MMAF cases (Fig. 3A).

### ICSI outcomes

**Fig. 4:**
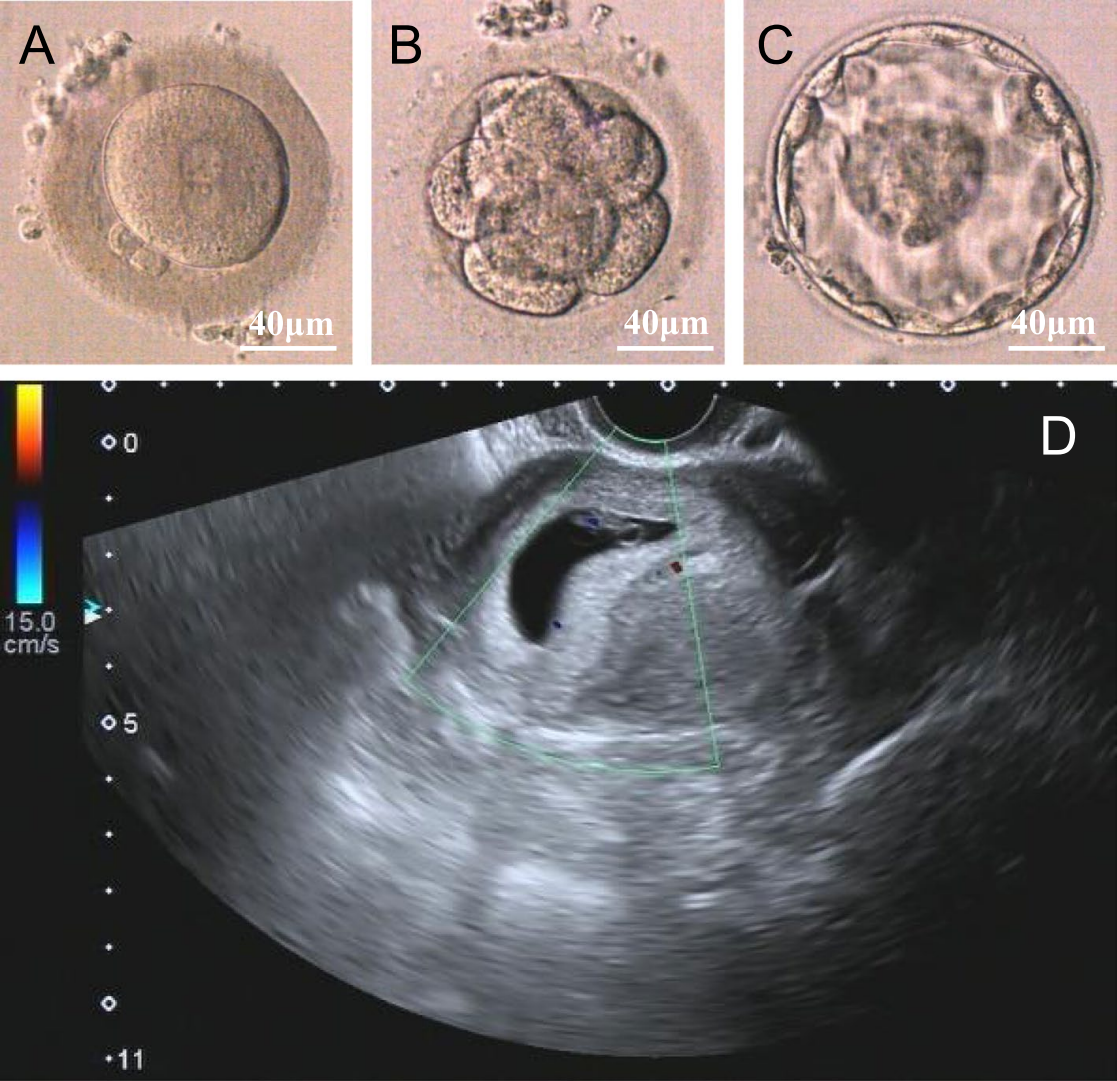
Typical morphology of the implanted embryo from F1 II-1 and his partner. High-quality blastocysts formed after standard embryo culture, and F1 II-1’ partner conceived successfully after the transfer of one frozen-thawed embryo. **A** :2 pronuclear fertilization; (**B**): 8-cell stage embryo; (**C**): blastocyst stage embryo; (**D**): the ultrasound image of gestational sac. Scale bar: 40 μm

ICSI treatment was performed due to male factor infertility in the two couples corresponding to the cases in the present study. All blastocysts were cryopreserved after standard embryo culture. Two months later, F1 II-1 and his partner successfully conceived after the transfer of one frozen-thawed embryo (Fig. 4). The F2 II-1 partner is currently waiting for embryo transfer. This result suggests that patients carrying *SPAG6* variants have a good prognosis for ICSI. The ICSI outcomes of the two couples are summarized in Supplementary Table 4.

## Discussion

Two patients with severe asthenoteratozoospermia harboring homozygous *SPAG6* variants were identified. The two cases presented a typical MMAF phenotype without other PCD-related symptoms. The expression level of SPAG6 protein was significantly lower in the spermatozoa of the two patients than in the controls, and IF analysis revealed that the fluorescent signal of SPAG6 was extremely weak and discontinuous in the sperm flagella of the patients. Taken together, these findings suggest that homozygous *SPAG6* gene variants are a novel causative genetic factor leading to nonsyndromic asthenoteratozoospermia with severe MMAF.

Motile flagella and their homologous structures, cilia, share an evolutionarily conserved axonemal structure consisting of nine peripheral microtubule doublets circularly organized around a central pair of microtubules (known as the “9 + 2” structure) [21]. Mutations in numerous genes encoding axoneme-associated proteins are closely tied to the improper assembly of cilia and flagella [6]. The human SPAG6 protein, a component of central microtubules, is essential for maintaining the structural stability of the axoneme. SPAG6 contains eight highly conserved armadillo-type repeats (ARMs), which mediate SPAG6 interactions with other central pair proteins [13]. The role of SPAG6 proteins in regulating flagellar and ciliary motility functions has been demonstrated in different biological models [14, 15, 22]. In *Chlamydomonas reinhardtii*, PF16, an axonemal protein orthologous to SPAG6, is localized to the central microtubule C1 of the axoneme, and is closely associated with flagellar motility [23]. The absence of PF16 causes instability of the C1 microtubule in the central pair, and flagellar paralysis [22]. *Spag6*-deficient mice are affected by hydrocephalus and infertility, suggesting that SPAG6 plays an important role in regulating cilial and flagellar motility [15]. Wu et al. [14]showed that *SPAG6* mutations in humans lead to a multi-systemic dysfunction phenotype, including chronic respiratory tract infections and male infertility. These findings highlight the important role of SPAG6 protein in the formation of cilia and flagella.

Unlike the studies mentioned above, the two cases carrying SPAG6 homozygous variants in the present study only presented with severe asthenoteratozoospermia. Considering that no other PCD-related symptoms were found in either case, invasive operations for obtaining ciliated cells, such as brushing or mucosal biopsy from the nose or trachea, were not performed, and it was therefore impossible to examine the morphology or ultrastructure of other ciliary tissue. However, no respiratory symptoms or visceral inversion were found upon physical examination and medical consultation, which was confirmed by lung CT screening. Therefore, we speculate that partial ciliary function was preserved in the patients.

Compared with individuals carrying mutations in *SPAG6* with typical PCD, the clinical phenotypes of the two probands were less severe in the present study. We speculate that this phenomenon may be attributed to a variety of factors: First, diverse mutation types and locations in some cilia-related genes may influence the severity of the phenotype in humans. For instance, the *DANH1* gene is a candidate gene for PCD, which encodes a core component of inner-arm heavy chain dynein, and an investigation carried out by Sha et al. [24] demonstrated that 12 patients harboring *DNAH1* variants only presented the MMAF phenotype in the absence of PCD-related symptoms. *DNAH9* is another candidate gene for PCD, but Tang et al. [25] reported that *DNAH9* variants can result in non-syndromic severe asthenospermia without PCD-related symptoms. Based on this, MMAF may be another form of classical PCD [6]. Second, according to animal models, the process of flagella formation is not identical to that of cilia. For instance, the Bbs4-null mouse model failed to form sperm flagella, but developed primary cilia in other organs normally [26]. Third, gene alternative splicing is widespread in mammals, and splicing variants usually display tissue-specific expression patterns [27]. Certain variants may affect the expression of SPAG6 in the testes rather than in other tissues. In addition, there may be other microtubule proteins that are similar to SPAG6 in phylogenesis, have similar functions, and may compensate for the absence of SPAG6 in other ciliated tissue.

Assisted fertilization with ICSI technology is the preferred option for MMAF patients because of the lack of sperm motility [6]. The potential risk of genetic defects is worthy of attention, apart from sperm morphological defects. Therefore, the female partners also underwent mutation screening for *SPAG6* before undergoing ICSI, and no deleterious mutations were found. After one cycle of frozen-thawed embryo transfer, the F1 II-1 couple successfully achieved clinical pregnancy. These results indicate that ICSI is an optimal management strategy for severe asthenoteratozoospermia induced by *SPAG6* variants.

## Conclusions

our findings expand the understanding of genetic defects in the *SPAG6* gene, which is a potential pathogenic factor for syndromic severe asthenozoospermia, such as PCD, and also for non-syndromic asthenoteratozoospermia with the MMAF phenotype. ICSI is recommended as an optimal strategy with a favorable prognosis for these patients.

## Supplementary Information

**Additional file 1:** **Supplementary Figure S1.** Typical PCD signs was excluded based on diagnostic imaging examination in F1 II-1and F2 II-1. (A) and (A’): The chest X-rays showed a normally located left-sided heart. (B) and (B’): The chest CT images showed normal lung and bronchus. (C) and (C’): The upper abdomen CT images excluded visceral inversion.

**Additional file 2.**

**Additional file 3.**

**Additional file 4.**

**Additional file 5.**

## Data Availability

The datasets utilized and/or analyzed in the study are available from the corresponding author on reasonable request.

## Abbreviations

MMAF: Multiple morphologic abnormalities of the flagella
PCD: Primary ciliary dyskinesia
WES: Whole exome sequencing
IF: Immunofluorescence
SEM: Scanning electron microscopy
TEM: Transmission electron microscopy
ICSI: Intracytoplasmic sperm injection
SIFT: Sorting Intolerant From Tolerant
