# Correction to: Homozygous SPAG6 variants can induce nonsyndromic asthenoteratozoospermia with severe MMAF

**DOI:** 10.1186/s12958-022-00939-w

**Published:** 2022-04-29

**Authors:** Chuan Xu, Dongdong Tang, Zhongmei Shao, Hao Geng, Yang Gao, Kuokuo Li, Qing Tan, Guanxiong Wang, Chao Wang, Huan Wu, Guanjian Li, Mingrong Lv, Xiaojin He, Yunxia Cao

**Affiliations:** 1 Reproductive Medicine Center, Department of Obstetrics and Gynecology, the First Affiliated Hospital of Anhui Medical University, No 218 Jixi Road, Hefei, 230022 Anhui China; 2 NHC Key Laboratory of Study On Abnormal Gametes and Reproductive Tract (Anhui Medical University), No 81 Meishan Road, Hefei, 230032 Anhui China; 3 Key Laboratory of Population Health Across Life Cycle (Anhui Medical University), Ministry of Education of the People’s Republic of China, No 81 Meishan Road, Hefei, 230032 Anhui China; 4 Anhui Province Key Laboratory of Reproductive Health and Genetics, No 81 Meishan Road, Hefei, 230032 Anhui China; 5 Biopreservation and Artificial Organs, Anhui Provincial Engineering Research Center, Anhui Medical University, No 81 Meishan Road, Hefei, 230032 Anhui China

**Correction to: Reprod Biol Endocrinol 20, 41 (2022)**

**https://doi.org/10.1186/s12958-022-00916-3**

Following publication of the original article [1], the authors reported an error in Fig. 3b wherein the labels F1 II-1 and F2 II-1 are interchanged. The correct Fig. 3 is presented below.

**Fig. 3:**
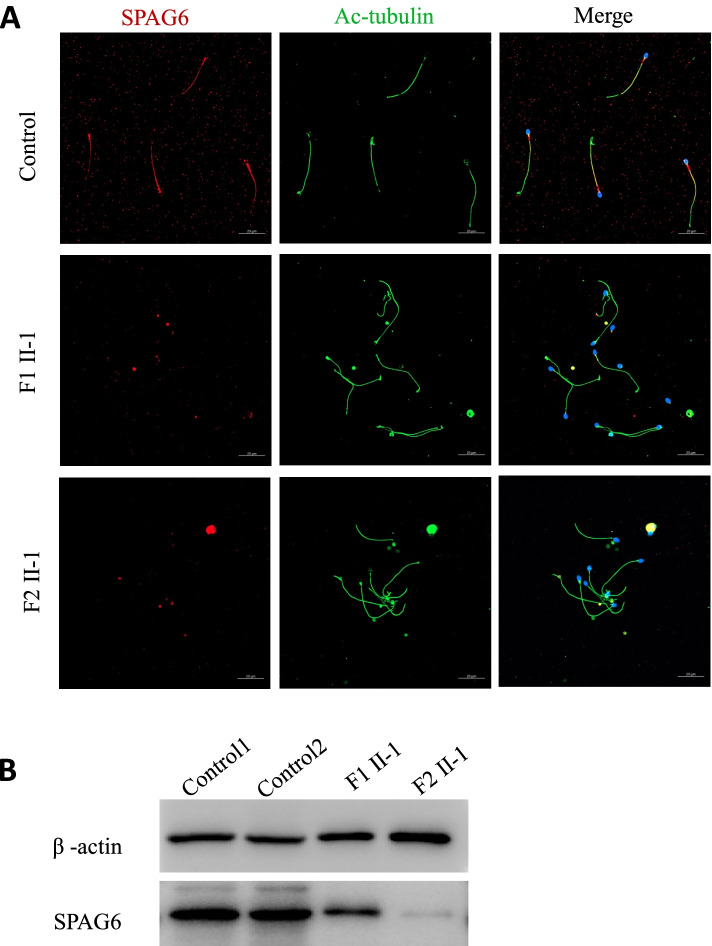
Lower expression of SPAG6 in spermatozoa from men harboring *SPAG6* variants. **A** Immunofluorescence analysis: SPAG6 staining (red) was located along entire the sperm flagella from a normal control, while SPAG6 staining was extremely weak and discontinuous in the sperm flagella from F1 II-1 and F2 II-1. The anti-acetylated tubulin staining (green) was used as a flagellar maker. Scale bar: 20 μm. **B** SPAG6 protein levels were determined using western blotting in spermatozoa from F1 II-1, F2 II-1 and two healthy controls. Beta-actin was used as loading control

The original article [1] has been updated.
