# Supplementary figures and images for: Homozygous SPAG6 variants can induce nonsyndromic asthenoteratozoospermia with severe MMAF
Source: Reprod Biol Endocrinol. 2022 Mar 1;20:41. doi: 10.1186/s12958-022-00916-3 (PMC8886842; doi:10.1186/s12958-022-00916-3)

## Slide 1
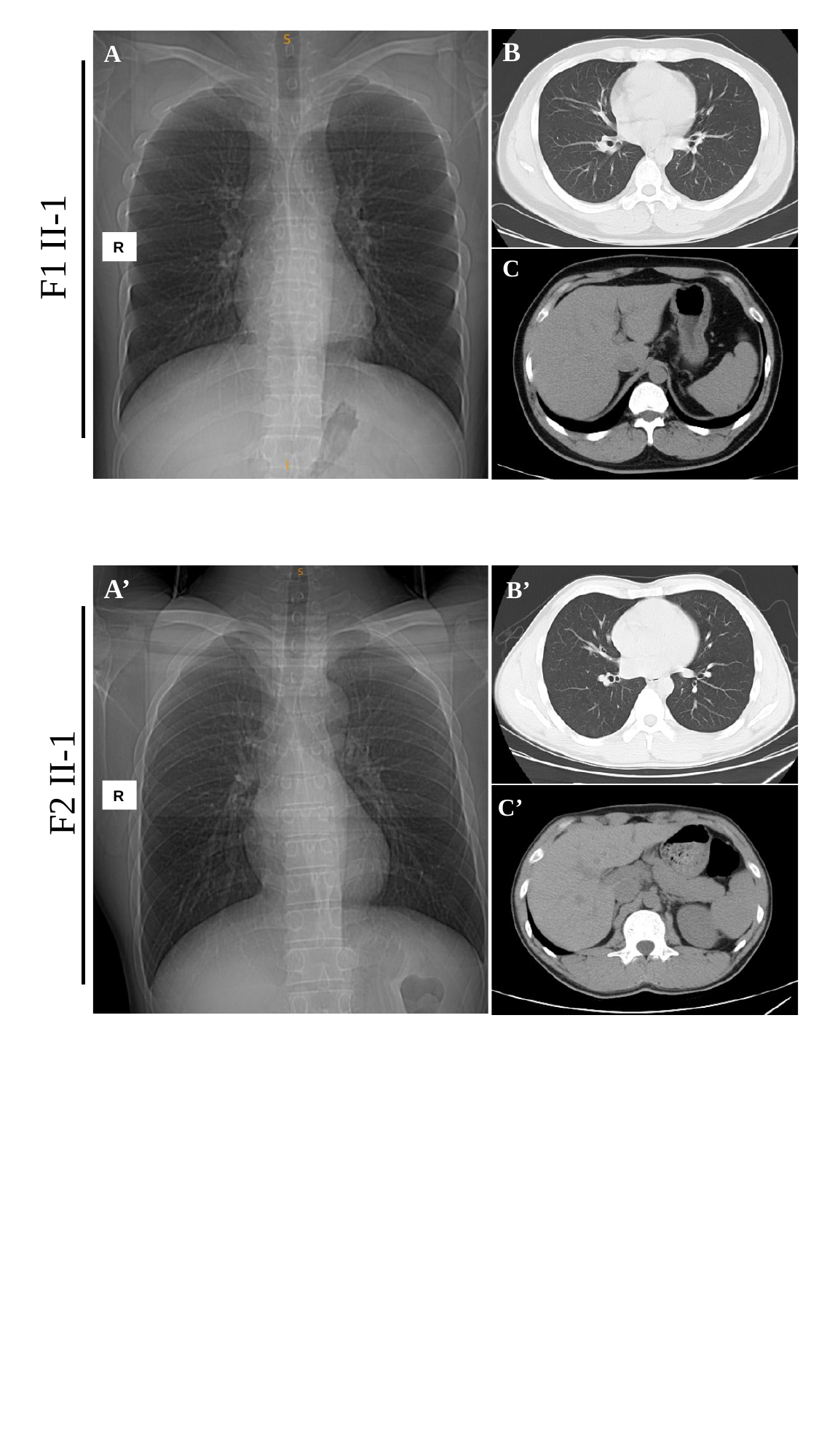

B
A
F1 II-1
R
C
A’
B’
F2 II-1
R
C’

Supplement: Supplementary file 1 — Additional file 1: Supplementary Figure S1. Typical PCD signs was excluded based on diagnostic imaging examination in F1 II-1and F2 II-1. (A) and (A’): The chest X-rays showed a normally located left-sided heart. (B) and (B’): The chest CT images showed normal lung and bronchus. (C) and (C’): The upper abdomen CT images excluded visceral inversion. [file 12958_2022_916_MOESM1_ESM.pptx]
