# Supplementary material for: Homozygous SPAG6 variants can induce nonsyndromic asthenoteratozoospermia with severe MMAF
Source: Reprod Biol Endocrinol. 2022 Mar 1;20:41. doi: 10.1186/s12958-022-00916-3 (PMC8886842; doi:10.1186/s12958-022-00916-3)
Supplement: Supplementary file 2 — Additional file 2. [file 12958_2022_916_MOESM2_ESM.docx]

| **Supplementary Table 1** Primers used for verification of *SPAG6* variants | | |
| --- | --- | --- |
| **Subject** | **Primer Names** | **Primer Sequences (5'-3')** |
| **F1 II-1** | M1-F | AAATGCAGGCTTCAGGGTAAAAC |
|  | M1-R | AAAACCCCACCTATGTTTTACTAAGC |
| **F2 II-1** | M2-F | GACCCACAGAACTGTCACAAGC |
|  | M2-R | TCAATTTAGCATCAGGGTTCAGG |
