# Supplementary material for: Homozygous SPAG6 variants can induce nonsyndromic asthenoteratozoospermia with severe MMAF
Source: Reprod Biol Endocrinol. 2022 Mar 1;20:41. doi: 10.1186/s12958-022-00916-3 (PMC8886842; doi:10.1186/s12958-022-00916-3)
Supplement: Supplementary file 4 — Additional file 4. [file 12958_2022_916_MOESM4_ESM.docx]

| **Supplementary Table 3** Clinical features of the patients carrying *SPAG6* variants | | |
| --- | --- | --- |
| **Subject** | **F1 II-1** | **F2 II-1** |
| **Age** | 29 | 26 |
| **PCD-related symptoms** | | |
| Otitis media | No | No |
| Rhinosinusitis | No | No |
| Wet cough | No | No |
| Bronchiectasis | No | No |
| Situs inversus | No | No |
| Congenital heart disease | No | No |
| Abbreviations: PCD: Primary ciliary dyskinesia. | | |
