# Supplementary material for: Homozygous SPAG6 variants can induce nonsyndromic asthenoteratozoospermia with severe MMAF
Source: Reprod Biol Endocrinol. 2022 Mar 1;20:41. doi: 10.1186/s12958-022-00916-3 (PMC8886842; doi:10.1186/s12958-022-00916-3)
Supplement: Supplementary file 5 — Additional file 5. [file 12958_2022_916_MOESM5_ESM.docx]

| **Supplementary Table 4** The clinical outcomes of ICSI treatment from men harbouring homozygous *SPAG6* variants | | |
| --- | --- | --- |
| **Subject** | **F1 II-1** | **F2 II-1** |
| Male age (year) | 29 | 26 |
| Female age (year) | 25 | 28 |
| ICSI cycles | 1 | 1 |
| No. of oocytes retrieved | 22 | 20 |
| Metaphase II stage oocytes | 16 | 13 |
| Oocytes fertilized | 14 | 10 |
| Blastocyst | 7 | 9 |
| High quality blastocyst | 6 | 9 |
| Embryos transferred | 1 | NA |
| Clinical pregnancy | Yes | NA |
| Abbreviations: ICSI: Intracytoplasmic sperm injection; NA: Not applicable; | | |
